# Supplementary material for: Factors associated with female age at first marriage: An analysis using all waves of the Pakistan Demographic and Health Survey
Source: PLoS One. 2022 Mar 15;17(3):e0264505. doi: 10.1371/journal.pone.0264505 (PMC8923498; doi:10.1371/journal.pone.0264505)
Supplement: Attachment — Submitted filename: Response to Reviewers-AFM Pakistan.docx [file pone.0264505.s001.docx]

**PONE-D-21-08432R1**

**Factors associated with female age at first marriage: an analysis using all waves of Pakistan Demographic and Health Survey**

**Response to Reviewers**

We thank the reviewers of the original version for their constructive and detailed comments. We list these below, point by point, with our responses.

***Reviewer #1***

*Line 70-71: Add % sign with all provincial estimates*

**We added the suggestion, (ll 75-76 in revised manuscript)**

*Line 156-159: “The reason behind this is interpretable, as, in later birth Cohorts, the current age is lower, so the females from these lower age groups have obviously lower mean AFM as compared to the females from earlier birth cohorts with the higher current age group.”*

*Age at first marriage doesn’t change with the increase in age of a particular birth cohort. The lower age groups may have lower AFM but it has no linkage with the current age. If a woman is first married at the age say 20, her AFM will remain at a constant value of 20 regardless of her current age at any point in time.*

*Line 159-163: “This could be the reason behind the scenario that we have not included current (age at the time of interview) age groups 15-19 and 20-24 in our analysis. As we are studying AFM, and our sample is only married women so if we include females having age groups at the time of interviews 15-19 and 20-24, then we cannot generalize our results to the whole of the female population.”*

*The sample of the study is currently married women at the time of survey and not the currently married women who were at their legal age at the time of first marriage. Even this case justifies exclusion of only under 18 category. A large fraction of females are married around their mid- twenties in Pakistan. So all females currently married at the time of survey should be included in the study to ensure generalization of results.

It is recommended to include the female belonging to age groups 15-24 in the analysis and update all tables and discussion accordingly.*

**We disagree with the reviewer’s suggestion. We are interested in measuring the secular change in the age at first marriage across birth cohorts.**  **However, in the Pakistan Demographic and Health Surveys (DHSs) only ever-married women aged 15-19 and 20-24 years old were interviewed. If, say, we include the women aged 15-19 years in our analysis, then all of them will report ages at marriage below 20 years, and their average age at marriage will be well below 20 years. The women in the same birth cohort—that is, who would have been aged 15-19 years at the date of the survey but who will marry at ages above 20 years will not be included in the sample because they were not interviewed. Because of this truncation of the distribution, our estimates of the mean age at first marriage for women aged 15-19 years at the time of each survey will be biased downwards, possibly severely. This can be illustrated by considering the women who were interviewed in the DHSs who were aged 15 years. All of these were married, else they would not have been interviewed, so they must all have married for the first time at age 15 years or less.**

**The same effect will be seen among women aged 20-24 years, though not so severely. However, since almost all women in Pakistan who will ever marry have done so by the time, they are aged 25 years, the bias should be negligible at ages 25 years and over. Hence, we only use data for women aged 25-29, 30-34, 35-39, 40-44, and 45-49 years at each survey in our calculations of the secular trend in the age at first marriage and in our analysis of the factors associated with the mean age at first marriage.**

**We have added a paragraph in the revised version of the paper to explain this point. (ll 117-127 in revised manuscript)**

***Reviewer #2***

*The main contribution of the study is that it studies the pattern of age at firm marriage (AFM) in Pakistan. It attempts to identify different demographic, social and cultural factors and examine their impact of AFM. This is an interesting topic however, following observations are needed to be addressed before acceptance.*

*1. The author should explicitly identify the objectives of the study.*

**We have added a sentence explicitly to identify these objectives (ll. 45-48 of the revised paper).**

*In addition, what methods have been adopted to meet the objectives?*

**The methods are described in ll. 107-64. We have made changes to the wording to make the description of the methods clearer.**

*2. In introduction part, although, the author gave references about the studies which examine the relationship between AFM and other variables. However, the study does not try to establish any theoretical relationship. It would be better if some theoretical connections are established in addressing the relationship.*

**We agree with the reviewer that a bit more is needed about the kinds of trends we might expect, and the covariates that we might expect to be associated with the age at first marriage, and we have added this (ll. 77-106). We do not think that a lengthy theoretical section on the determinants of age at first marriage in Pakistan is appropriate for this paper, as our intention is empirically to analyse trends in the mean age at first marriage and in the factors associated with age at first marriage in the country with a view to understanding the likely impact of changes in the age at first marriage on fertility trends.**

*3. On page 7, line 97-99, the author mentioned that there are more than twenty ethnic groups in Pakistan and the researcher divided these groups into eight categories. The author did not specify the criteria how these categories have been merged.*

**In the Pakistan Demographic and Health Surveys, direct questions on ethnicity were not asked. In 1990-91 there is no variable in the data from which ethnicity can be easily inferred. For the surveys of 2006-07 and 2012-13 we have inferred ethnicity from answers to the question ‘What is your mother tongue?’ The possible answers were in 2006-07, Urdu, Punjabi, Sindhi, Pushto, Balochi, English, Barauhi, Siraiki, Hindko, Kashmiri, Parahi, Potowari, Marwari, Farsi and other; in 2012-13 additional categories Shina, Brushaski, Wakhi, Chitrali/Khwar and Balti were added. In the present study, we reduced these to eight categories by taking the seven largest linguistic groups as individual categories and combining the remainder into a residual ‘others’ category. We added (ll. 149-56)**

*4. In table 5, the author showed the results of multiple regression model however, no regression equation has been presented in material and method part. It would be appropriate if the author develop a regression equation which incorporates both dependent and independent variables.*

**We thank you for this suggestion. We have added a regression equation (ll. 260-69).**

*Moreover, the value of R-square and F-Statistic should also be mentioned in the table 5.*

**We thank you for this suggestion. We have added the value of R-square and F-Statistic for regression in the table 5.**

**Finally, we asked a native English speaker who is also the editor of an academic journal in the English language to copy-edit the paper. The result has been a thorough revision of the language in the text.**
