# Supplementary material for: Factors associated with female age at first marriage: An analysis using all waves of the Pakistan Demographic and Health Survey
Source: PLoS One. 2022 Mar 15;17(3):e0264505. doi: 10.1371/journal.pone.0264505 (PMC8923498; doi:10.1371/journal.pone.0264505)
Supplement: Attachment — Submitted filename: Response to editor and Reviewers-Second Revision.docx [file pone.0264505.s002.docx]

PONE-D-21-08432R1

Factors associated with female age at first marriage: an analysis using all waves of the Pakistan Demographic and Health Survey (revised version)

Response to editor and reviewers

___________________________________________________________________________________________

The reviews of the revised version of the paper made only one comment. This concerns the decision not to include analysis of the women aged 15-24 years for the 2012-2013 and 2017-2018 Demographic and Health Surveys. The Reviewer disagreed with this decision. In our response with the first revision, we explained that one of our purposes is to analyse the trend in the age at first marriage in Pakistan over time. If we include the women aged 15-19 years in our analysis all of them will report ages at marriage below 20 years, and their average age at marriage will be well below 20 years. The women in the same birth cohort—that is, who would have been aged 15-19 years at the date of the survey but who will marry at ages above 20 years will not be included in the sample because they were not interviewed.

The Reviewer wrote:

‘This argument is not convincing. The generalization of results in any survey-based study is restricted to the particular sample selected for the survey. Since the analysis is over time, the claimed inclusion of females who decide to marry above say age 20 is adequately presented in the subsequent survey. Even if this argument is accepted for the age bracket 15-19 [years], it is totally unacceptable for age 20-24. The study itself claims in l. 237 that “In general, more than 90% of the females marry before the age of 25 years”, so inclusion of this age bracket is very less likely to create any downward bias in the estimates.’

We disagree with the Reviewer on two counts: (1) about the generalisation of our results; and (2) about the downward bias in the estimates.

(1) The point made by the Reviewer is true in the sense that we cannot make inferences out of sample. However, we are not trying to do this. We are wanting to analyse the factors associated with the age at marriage among groups of women who have had every chance to get married. We also want to compare results from the 2017-2018 and 2012-2013 surveys with those in analyses of earlier surveys which only used data for women aged 25-49 years. In order to achieve comparability and to analyse only women who have had every chance to get married, we restrict attention to women aged 25-49 in the 2012-2013 and 2017-2018. We accept that this reduces our sample size, and it also means that we cannot make inferences or generalisations about women who were aged below 25 years in 2017-2018, but this is a price we are happy to pay.

(2) We can demonstrate that the inclusion of women aged 15-19 years and 20-24 years results in a downward bias in our estimate of the trend. Our data come from four separate Demographic and Health Surveys, taken in 1990-1991, 2006-2007, 2012-2013 and 2017-2018. In each of these surveys we have data for a set of age cohorts and for each age cohort we can calculate the mean age at marriage. We can also calculate the average calendar year in which the women in each age cohort in each survey married. For example, the 2017-2018 survey took place over the period November 2017 – April 2018. On average, therefore, women were interviewed around the end of January 2018. The women aged 25-29 years in this survey were, on average, aged 27.5 years at the end of January 2018, and hence they were born, on average, at the end July 1990. They had an average age at marriage of 19.43 years, meaning that they married, on average, at the beginning of 2010. We can similarly calculate the average date of marriages for other age cohorts in other surveys. Plotting the average age at marriage of each age cohort against the average date of the relevant marriages allows us to track the trend over time in the mean age at marriage. The results are shown for all four surveys in the diagram below.

The black circles denote ages at marriage computed from data for women aged 25-49 years in the four surveys. The squares denote ages at marriage computed from data for women aged 20-24 and 15-19 years in the 2012-2013 and 2017-2018 surveys. It is clear that using data for woman aged 20-24 and 15-19 years will create a downward bias in our estimate of the trend in the age at marriage in recent years. The downward bias is, as the Reviewer correctly points out, less severe for women aged 20-24 years than for women aged 15-19 years, but it is nevertheless present for both age groups.

Age at marriage in Pakistan: estimates from four Demographic and Health Surveys

Average age at marriage

| Year of marriage | 25-49 years | 15-24 years |
| --- | --- | --- |
| 1990.14 | 19.54 |  |
| 1995.04 | 19.44 |  |
| 2000.53 | 19.93 |  |
| 2005.55 | 19.95 |  |
| 2010.03 | 19.43 |  |
| 2013.76 | 0 | 18.16 |
| 2016.72 | 0 | 16.12 |
| 1984.38 | 18.78 |  |
| 1989.45 | 18.85 |  |
| 1994.77 | 19.17 |  |
| 2000.18 | 19.58 |  |
| 2004.87 | 19.27 |  |
| 2008.67 | 0 | 18.07 |
| 2011.8 | 0 | 16.2 |
| 1978.23 | 18.93 |  |
| 1982.97 | 18.67 |  |
| 1987.92 | 18.62 |  |
| 1992.97 | 18.67 |  |
| 1998.03 | 18.73 |  |
| 1963.13 | 19.43 |  |
| 1967.21 | 18.51 |  |
| 1972.17 | 18.47 |  |
| 1976.65 | 17.95 |  |
| 1981.46 | 17.76 |  |

We have not included a lengthy discussion of this issue in the second revision of our paper as the existence of these selection effects and the problems they can cause is a standard and well-known feature of the analysis of retrospective survey data (see, for example, the discussion in A. Hinde, *Demographic Methods*, London, Arnold, 1998, pp. 135-6).

However, we have made the following changes to the paper to try to explain why we do not analyse data for women aged under 25 years at the time of the 2012-2013 and 2017-2018 surveys:

- we have added a new Table 2 which includes estimates of the mean age at marriage for women aged 15-19 and 20-24 years in the 2012-2013 and 2017-2018 surveys
- we have renumbered the subsequent tables
- we have added a discussion of the results in the new Table 2 as follows:

‘Table 2 shows the mean age at first marriage for currently married women in the four surveys according to the age of the women at the time of the survey. The mean age at marriage for women in the 2017-18 survey was between 19.4 and 20.0 years for all age cohorts over 25 years. For younger age cohorts the mean age at marriage was substantially lower (18.16 years for those aged 20-24 years and only 16.12 years for those aged 15-19 years). These lower ages at marriage for the younger age cohorts are selection effects. Women aged 15-19 years who report an age at marriage in the Demographic and Health Surveys must all have married at ages less than 20 years; those women in the same cohort who are yet to marry will not report an age at marriage. As a consequence, the estimates of the mean age at marriage for this age cohort based on the data in the survey are underestimates of the mean age at marriage that would be reported by the women in the same age cohort were they to be interviewed when those who will eventually marry have all married. The same is true to a lesser extent for women aged 20-24 years. but not for older women, as the vast majority of women in Pakistan will marry before their 25th birthdays. Because of this, we exclude women aged under 25 years from the analysis which follows.’

- Because we now discuss the mean ages at marriage for women in different age cohorts at an earlier point in the paper, it made sense to us to move Figure 2 which examines the time trend so that it comes before Figure 1. We have accordingly done this, and renumbered these figures accordingly.
